# Tobacco use and associated factors among Adults in Uganda: Findings from a nationwide survey

**DOI:** 10.1186/s12971-016-0093-8

**Published:** 2016-08-11

**Authors:** Steven Ndugwa Kabwama, Sheila Ndyanabangi, Gerald Mutungi, Ronald Wesonga, Silver K. Bahendeka, David Guwatudde

**Affiliations:** 1 Uganda Public Health Fellowship Program, Field Epidemiology Track, Ministry of Health, Kampala, Uganda; 2 Mental Health and Substance Abuse, Ministry of Health, Kampala, Uganda; 3 Control of Non-Communicable Diseases Desk, Ministry of Health, Kampala, Uganda; 4 School of Statistics and Planning, Makerere University College of Business and Management Sciences, Kampala, Uganda; 5 St. Francis Hospital, Nsambya, Kampala, Uganda; 6 Department of Epidemiology & Biostatistics, School of Public Health, Makerere University College of Health Sciences, Kampala, Uganda

**Keywords:** Tobacco use, Non-communicable diseases, WHO STEPs methodology, Sub-Saharan Africa, Uganda

## Abstract

**Background:**

Tobacco use and the exposure to tobacco smoke is one of the most preventable causes of death and disability globally. The risk is even higher among daily tobacco users. The World Health Organization (WHO) has recommended that surveillance of major risk factors for Non Communicable Diseases (NCDs) such as tobacco use is imperative to predict the future burden of NCDs, identify interventions to reduce future burden and monitor emerging patterns and trends. In 2014 the first Uganda nation-wide NCD risk factor survey was carried out to estimate the prevalence of major NCD risk factors. We analyzed data from this survey to estimate the prevalence of daily tobacco use and associated risk factors.

**Methods:**

A nationally representative sample was drawn stratified by the four regions of the country. The WHO’s STEPwise tool was used to collect data on demographic and behavioral characteristics including tobacco use, physical and biochemical measurements. Tobacco use was divided into three categories; daily tobacco use, daily smoked tobacco use and daily smokeless tobacco use. Weighted logistic regression analysis was used to identify factors associated with daily tobacco use.

**Results:**

Of the 3983 participants, 9.2 % (366) were daily tobacco users, 7.4 % (294) were daily smoked tobacco users and 2.9 % (115) were daily smokeless tobacco users. Male participants were more likely to be daily tobacco users compared with female participants AOR 5.51 [3.81–7.95]. Compared with participants aged 18–29 years, those aged 30–49 years were more likely to be daily tobacco users AOR 2.47 [1.54–3.94] as were those aged 50–69 years AOR 2.82 [1.68–4.74]. Compared with participants without any education, those with primary education were less likely to be daily tobacco users AOR 0.43 [0.29–0.65], as were those with secondary education AOR 0.21 [0.14–0.33] and those with university level of education AOR 0.23 [0.11–0.48]. Compared with participants in the central region, those in the eastern region were more likely to be daily tobacco users AOR 2.14 [1.33–3.45] as were those in the northern region AOR 4.31 [2.79–6.45] and those in the western region AOR 1.87 [1.18–2.97]. Participants who were underweight were more likely to be daily tobacco users compared with people with normal BMI AOR 2.19 [1.48–3.24].

**Conclusions:**

In agreement with previous surveys on tobacco use, there is a high prevalence of tobacco use in Uganda with almost 1 in every 10 Ugandans using tobacco products daily**.** Being older, male, having no formal education, residing in the east, north and western regions and having low BMI were significantly associated with daily tobacco use. This information provides a useful benchmark to the National Tobacco Control Program for the designing of public health interventions for the control and prevention of tobacco use in Uganda.

**Electronic supplementary material:**

The online version of this article (doi:10.1186/s12971-016-0093-8) contains supplementary material, which is available to authorized users.

## Background

Tobacco use and the exposure to tobacco smoke is one of the most preventable causes of death and disability globally. The risk of disability is even higher among daily tobacco users [1]. In fact, compared to light smokers, heavier smokers have been shown to have an increased risk of ischemic stroke [2] and serum lipid and lipoprotein concentrations [3]. Projections indicate that between 2002 and 2030, mortality related to tobacco use and its exposure is expected to reduce by 9 % in high income countries but double from 3.4 million to 6.8 million in low and middle income countries [4]. The World Health Organization (WHO) Global Status report on Non Communicable Diseases (NCDs) indicated that in Uganda, tobacco use was a major risk factor for NCDs which account for 25 % of all deaths in the country [5]. Statistics from the Uganda Cancer Institute also indicate that 25 % of lung cancer patients were tobacco users and 16 %, 13.7 % and 12.6 % of oral, stomach and throat cancer patients were former tobacco users [6]. The WHO has recommended that surveillance of the major risk factors for NCDs such as tobacco use is imperative to predict the future burden of NCDs, identify interventions to reduce future burden and monitor emerging patterns and trends [7]. Currently the surveillance of tobacco use among adults in Uganda is done through quinquennial Uganda Demographic and Health Surveys (UDHS) [8–11] as well as the Global Adult Tobacco survey [12]. The UDHS provides national data on demographic and health characteristics and contains one question on tobacco use. However it only reports prevalence, does not relate the prevalence to social and demographic characteristics and does not assess magnitude of risk in terms of daily tobacco use. The GATS provides information on adult tobacco use and key tobacco control indicators such as tobacco advertising, exposure to secondhand smoke and the economics of tobacco smoking but does not assess physical and biochemical outcomes. In 2014 the first nation-wide NCD risk factor survey was carried out in Uganda to estimate the prevalence of major NCD risk factors. We analyzed data from this survey to estimate the prevalence of daily tobacco use and demographic, physical and biochemical assessments associated with it.

## Methods

A cross-sectional study design was used to conduct the survey between March and July 2014. The survey used the WHO’s standardized tool for analysis of risk factors for NCDs [7]. A detailed description of the survey design, study population and sampling procedures have been described elsewhere [13, 14]. Here, we focus on the sections of the methodology that are relevant to this paper.

### Measurements

In STEP 1, the tool collected demographic, socio-economic and behavioral characteristics including tobacco use. Level of education was assessed in terms of number of formal school years completed. The first 7 years of formal school are referred to as primary school, followed by 6 years of secondary school and at least 3 years of University education. Smoked tobacco products included those whose use involves combustion of the tobacco product and inhalation of tobacco smoke through the mouth while smokeless tobacco products are those whose use involves chewing the tobacco product or sniffing it through the mouth or nose. Tobacco users included participants who self-reported to have used smoked tobacco products such as cigarettes, cigars, shisha or pipes and those who self-reported to have used smokeless tobacco products such as Bugolo, Taba, Etabe, Kuba or Gutka. Daily tobacco users included participants who self-reported to have used any smoked tobacco products daily and those who self-reported to have used any smokeless tobacco products every day. Participants were also asked how many products they smoke daily and how many times a day they used smokeless tobacco products.

In STEP 2, weight, height and blood pressure were measured. Blood pressure measurements were taken on the left arm using battery powered digital blood pressure machine (Boso Medicus Uno**®**). Three readings were taken 3–5 min apart.

After measuring height, weight and blood pressure, a blood sample was collected to measure fasting plasma glucose using a CardioChek® PA meter. This was done on the day following the interview and physical measurements. Participants had to report compliance with an overnight 8-h fast. Participants who engaged in physical exercise and smoking were ineligible for collection of a blood sample.

### Statistical analysis

Considering that approximately 43 % of adults in Uganda are aged 18 years or older [9], participants were categorized in age-groups of 18–29, 30–49 and 50–69. These categories have also been used elsewhere in analyses of national data in Uganda [13, 14].

Participants were classified as having raised blood pressure if the average of the last two systolic blood pressure readings was at least 140 mm Hg and/ or the average of the last two diastolic blood pressure readings was at least 90 mm Hg [15]. Participants who were on medication for high blood pressure at the time of assessment were also classified as having raised blood pressure.

To identify factors associated with daily tobacco use, weighted logistic regression analysis was used to estimate both crude and adjusted odds ratios (AORs) and their corresponding 95 % confidence intervals (CIs). We identified factors that could potentially be associated with daily tobacco use and included these as independent variables in a model. The independent variables included in the model were sex, age, level of education, marital status, employment, geographical region of residence, urban or rural residence, Body Mass Index (BMI), blood pressure and fasting plasma glucose. We then used stepwise backward elimination to discard those least significantly associated with daily tobacco use. Variables were discarded one at a time starting with the one with the largest p-value. Variables were retained in the model if they achieved a 5 % level of statistical significance (α = 0.05). The sampling selection weights were used. All statistical analyses were performed using STATA version 12.

## Results

### Characteristics of participants

Out of the 4900 targeted sample, 3987 subjects took part in the survey giving a participation rate of 81.4 %. Of the 3987 participants that participated in the survey, 3983 provided information on their tobacco use and are included in this analysis. Of the 3983 participants, 2382 (59.8 %) were females, 1678 (42.1 %) were aged 30–49 years and 2642 (66.3 %) were either married or cohabiting (Table 1). Among these participants, 2600 (65.3 %) were engaged in some form of employment, 1084 (27.2 %) lived in urban areas and 652 (16.4 %) did not have any form of education.

**Table 1:** Characteristics of participants

Characteristic	-n-	Summary measure (%)
Sex		
Male	1601	40.2
Female	2382	59.8
Age		
18–29	1616	40.6
30–49	1678	42.1
50–69	689	17.3
Level of education^a^		
No formal school	652	16.4
Primary	1625	41.0
Secondary	1317	33.2
University or higher	374	9.4
Ethnic group		
Baganda	775	19.5
Banyankore/Bakiga	705	17.7
Karimojong/Acholi	281	7.1
Basoga	316	7.9
Bagisu/ Iteso	453	11.4
Lugbara/Madi	249	6.3
Other	1204	30.2
Marital status		
Never married	627	15.7
Currently married/cohabiting	2642	66.3
Other (separated or divorced or widowed)	714	17.9
Employment status^a^		
Employed	2600	65.3
Unemployed	1382	34.7
Region		
Eastern	1292	32.4
Central	963	24.2
Northern	779	19.6
Western	949	23.8
Residence		
Urban	1084	27.2
Rural	2899	72.8

^a^The totals do not add up to 3983 because some observations were not stated

### Distribution of tobacco use

The results of the survey showed that of the 3983 participants, 9.2 % (366) were daily tobacco users, 7.4 % (294) were daily smoked tobacco users and 2.9 % (115) were daily smokeless tobacco users. Of the daily tobacco users, 80.3 % (294/366) used smoked tobacco products daily while 31.4 % (115/366) used smokeless tobacco products daily. Among participants who used smoked tobacco products daily, 79.3 % (233) were males, 51.4 % (151) were aged 30–49 years and 76.5 % (225) were from rural areas (Table 2). Among participants reporting use of smokeless tobacco products daily, 58.3 % (67) were females, 40.9 % (47) were aged 50–69 years and 94.9 % (108) were from rural areas.

**Table 2:** Categorisation of daily tobacco use by sex, age and urban-rural residence

	Smoked *n* = 294 (%)	Smokeless *n* = 115 (%)	Smoked and smokeless *n* = 366 (%)
Sex			
Male	233 (79.3)	48 (41.7)	255 (69.7)
Female	61 (20.7)	67 (58.3)	111 (30.3)
Age			
18–29	44 (15.0)	25 (21.7)	61 (16.7)
30–49	151 (51.4)	43 (37.4)	180 (49.2)
50–69	99 (33.6)	47 (40.9)	125 (34.1)
Residence			
Urban	69 (23.5)	7 (6.1)	72 (19.7)
Rural	225 (76.5)	108 (94.9)	294 (80.3)

Among participants who use smoked tobacco products daily, 62.2 % (183) use manufactured cigarettes while 39.8 % (117) use hand rolled cigarettes (Table 3). The average number of manufactured cigarettes smoked daily is 4.7 ± 4.6 (1–40) while the average number of hand-rolled cigarettes smoked daily is 5.0 ± 4.3 (1–21). Among participants who use smokeless tobacco products, 70.4 % (81) use the product by sniffing through the mouth while 24.3 % (28) use smokeless tobacco product by chewing them. The average number of times a participant used a smokeless product by sniffing through the mouth was 4.8 ± 3.4 (1–12) while the average number of times a participant used a smokeless tobacco product by chewing was 4.0 ± 3.3 (1–12).

**Table 3:** Categorization of tobacco users by frequency and intensity of tobacco use

Tobacco product	Daily users	Average daily use
Smoked tobacco products	*n* = 294 (%)	Number of products/day ± SD (range)
Manufactured cigarettes	183 (62.2)	4.7 ± 4.6 (1–40)
Hand-rolled cigarettes	117 (39.8)	5.0 ± 4.3 (1–21)
Pipes full of tobacco	43 (14.6)	3.2 ± 3.2 (1–12)
Cigars, cheroots, cigarillos	5 (1.7)	17 ± 20.3 (1–50)
Shisha	2 (0.7)	25.5 ± 34.6 (1–50)
Other	12 (4.1)	6.9 ± 13.9 (1–50)
Smokeless tobacco products	n = 115 (%)	Number of times/day
Snuff by mouth (taba/etabe)	81 (70.4)	4.8 ± 3.4 (1–12)
Snuff by nose (Bugolo)	17 (14.8)	3.6 ± 2.6 (1–10)
Chewed tobacco (gutka, Kuba)	28 (24.3)	4.0 ± 3.3 (1–12)
Other	2 (1.7)	3.5 ± 3.5 (1–6)

### Factors associated with tobacco use

The factors found to be significantly associated with daily tobacco use were sex, age, level of education, marital status, geographical region and Body Mass Index (BMI). The odds of being a daily tobacco user were significantly higher for males compared to females AOR 4.53 [3.21–6.40] (Table 4). Compared with participants aged 18–29 years, those aged 30–49 years were more likely to be daily tobacco users AOR 2.96 [1.93–4.52] as were those aged 50–69 years AOR 3.82 [2.42–6.03]. Compared with participants without any form of education, those with primary education were less likely to be daily tobacco users AOR 0.46 [0.31–0.68], as were those with secondary education AOR 0.21 [0.13–0.32] and those with university level of education AOR 0.21 [0.10–0.42]. Compared with participants in the central region, those in the eastern region were more likely to be daily tobacco users AOR 2.24 [1.40–3.59] as were those in the northern region AOR 4.21 [2.74–6.48] and those in the western region AOR 1.82 [1.14–2.88]. Compared with participants with normal body mass index (BMI), persons who were overweight or obese were less likely to be daily tobacco users AOR 0.64 [0.38–1.08]. Persons who were underweight were more likely to be daily tobacco users compared with people of normal BMI AOR 2.31 [1.57–3.40].

**Table 4:** Crude and adjusted odds ratios (ORs) of being a daily tobacco user compared to not being a daily tobacco user

	-n-	Number of daily tobacco users (%)	Crude OR [95 % CI]	Adjusted OR^a^ [95 % CI]
Sex				
Female	2382	111 (4.7)	1.0	1.0
Male	1601	255 (15.9)	5.33 [3.66–7.79]	5.51 [3.81–7.95]
Age				
18–29	1616	61 (3.8)	1.0	1.0
30–49	1678	180 (10.7)	2.37 [1.43–3.94]	2.47 [1.54–3.94]
50–69	689	125 (18.1)	2.90 [1.68–5.01]	2.82 [1.68–4.74]
Education				
No formal school	652	117 (17.9)	1.0	1.0
Primary	1625	164 (10.1)	0.45 [0.30–0.67]	0.43 [0.29–0.65]
Secondary	1317	65 (4.9)	0.19 [0.12–0.31]	0.21 [0.14–0.33]
University or higher	374	19 (5.1)	0.23 [0.10–0.49]	0.23 [0.11–0.48]
Employment				
Employed	2600	242 (9.3)	1.0	1.0
Unemployed	1382	124 (9.0)	1.33 [0.88–2.02]	1.35 [0.91–2.00]
Region				
Central	963	52 (5.4)	1.0	1.0
Eastern	1292	90 (7.0)	2.29 [1.36–3.86]	2.14 [1.33–3.45]
Northern	779	131 (16.8)	4.54 [2.87–7.16]	4.31 [2.79–6.45]
Western	949	93 (9.8)	2.20 [1.31–3.68]	1.87 [1.18–2.97]
Residence				
Urban	1084	72 (6.6)	1.0	1.0
Rural	2899	294 (10.1)	0.83 [0.55–1.25]	0.81 [0.56–1.18]
BMI (kg/m^2^)				
Normal 18.5–24.9	2531	255 (10.2)	1.0	1.0
Overweight/obese ≥25	828	44 (7.6)	0.71 [0.41–1.24]	0.64 [0.38–1.09]
Underweight <18.5	330	84 (25.5)	2.17 [1.46–3.22]	2.19 [1.48–3.24]
Blood pressure (mmHg)				
Normal	3617	539 (14.9)	1.0	1.0
Raised or medication	366	57 (15.6)	0.87 [0.56–1.36]	0.85 [0.56–1.31]
FPG (mmol/L)				
< 6.1	3562	322 (9.0)	1.0	1.0
6.1–6.9	82	5 (6.1)	0.52 [0.12–2.29]	0.48 [0.11–2.19]
> =7 or on DM Rx	46	6 (13.0)	1.81 [0.62–5.30]	1.89 [0.64–5.58]
Marital status				
Never married	627	36 (5.7)	1.0	1.0
Currently married	2642	231 (8.7)	1.57 [0.86–2.87]	1.31 [0.77–2.26]
Other (separated/divorced/widowed)	714	99 (13.9)	2.81 [1.41–5.61]	2.54 [1.36–4.72]

^a^Adjusted for sex, age, education, region, marital status and BMI

## Discussion

The analysis revealed a daily tobacco use prevalence of 4.7 % among women, 15.9 % among men and overall prevalence of 9.2 %. These findings are in agreement with the Global Adult Tobacco Survey 2013 (4.6 % women, 11.6 % men) [12] and Uganda Demographic and Health Survey (UDHS) 2011 (3 % women, 15 % men) [8]. The findings particularly among males however are lower in comparison to the findings in the UDHS 2001 (3 % women and 25 % men) [11] and UDHS 2006 (4 % women and 23 % men) [10]. The lower prevalence reported in this survey could be due to the narrower definition of tobacco use where only daily tobacco users were considered while occasional tobacco users were excluded. In comparison with Uganda with an overall daily tobacco use prevalence of 9.2 %, the Democratic Republic of Congo (DRC) (4.4 %) [16], Ethiopia (4.6 %) [17] and Zambia (5.0 %) [18] reported lower prevalence while Tanzania (15.9 %) [19] and Mozambique (16.7 %) [20] reported higher prevalence of tobacco use. However besides the survey done in Tanzania, the other surveys assessed only daily use of smoked tobacco products and excluded smokeless tobacco products.

The results of the analysis revealed that of the 366 daily tobacco users, 80.3 % used smoked tobacco products daily and 31.4 % used smokeless tobacco products daily. This finding has important public health and policy implications to direct efforts to reducing access to and use of smoked tobacco products in comparison with smokeless tobacco products. In addition, 62.2 % of daily smokers used manufactured cigarettes. This finding implies that policy adjustments such as tax increases on tobacco could help in reducing access to and use of manufactured tobacco products especially to youth and young adults as has been demonstrated elsewhere [21].

Daily smoked tobacco product users were mostly men (79.3 %) and rural dwellers (76.5 %) in contrast with daily smokeless tobacco product users who were mostly women (58.3) and rural dwellers (94.9). This finding is similar to the GATS 2013 [12] where more males (10.3 %) than females (1.8 %) used any smoked product while more females (3 %) than males (1.7 %) used smokeless tobacco products. The high prevalence of tobacco use in rural areas could be due to easier access to the tobacco products in the areas especially when the rural area relies economically on the tobacco that is grown there [22]. Also, the smokeless tobacco use by women and rural dwellers could be because it is cheaper [23] while others may consider its use it as a harm reduction strategy [24, 25] in comparison with smoked tobacco.

The association analysis revealed statistically significant associations between daily tobacco use and age, sex, geographical region, level of education and BMI. The association between age and tobacco use as well as sex and tobacco use is no surprise finding and has been reported in other surveys [8, 10–12] in Uganda where males and older persons are more likely to use tobacco products. Studies done in the US found that among men, health behaviors such as tobacco use were predicted by masculinity and perceived normativeness of other men’s behaviors [26], social desirability and differences in nicotine sensitivity [27]. Qualitative investigations are required to decipher the motivations towards tobacco use among men in Uganda compared to women. The association between region and tobacco use where people from other regions besides the central region are more likely to use tobacco, age and tobacco use where older people are more likely to use tobacco, and education level and daily tobacco use where more educated people are less likely to use tobacco might be an indication of a wider socio-economic context within which tobacco use is enshrined [28]. Western, Eastern and Northern regions have been reported to have higher levels of poverty compared to the Central region [29]. The elderly in Uganda have been shown to experience chronic poverty [30]. Education has also been described as an index of socio-economic circumstances in life [31] such that the tobacco use among older people, persons in the Northern, Eastern and Western regions and people with low education levels is a reflection of an environment of poverty, stress and economic disadvantage all of which not only foster smoking but have also been shown to discourage cessation [32]. Tobacco control efforts should go beyond limiting access to and use of tobacco products but encompass efforts aimed at improvement of socioeconomic status and standard of living in general especially in rural areas. With the tobacco industry shifting its focus from North America and Europe to Africa and Asia [33], this information will provide evidence for the formation of policies that integrate tobacco control with socio-economic development in developing countries in Africa and Asia. The inverse association between BMI and tobacco use is of particular interest. A similar association has been found elsewhere [34, 35] although other studies have found smoking and BMI to be positively associated [36]. The explanation for the inverse association is that nicotine—the active component in tobacco leads to an increase in energy expenditure and subsequent loss of appetite which explains why smokers are leaner than non-smokers [37, 38]. Tobacco use cessation programs should entail components aimed at improving nutritional status of smokers in Uganda.

### Strengths and limitations

A limitation of this analysis is that the use of the variable “daily tobacco use” excludes occasional tobacco users and thus understates the magnitude of tobacco use among Ugandans. However the alternative variable in the WHO STEPS questionnaire was “current tobacco use” which is not time-bound, vague and subject to the interpretation of the interviewee. Future STEPS surveys should include a time component in the assessment of “current tobacco use”. The other limitation of the analysis is that because of the cross sectional nature of the survey, inferences of causal relationships between tobacco use and the other independent variables need to be made with caution. The observed association between tobacco use and BMI for example could be that lean persons were the ones using tobacco and not vice versa. In addition, the assessment of the primary outcome variable “daily tobacco use” was based on self reports which could introduce bias. The sample selection however was systematic enough for the findings to be generalizable to the Ugandan population. Also the use of a standardized questionnaire means findings can be compared to those from other countries.

## Conclusion

In agreement with previous surveys on tobacco use, there is a high prevalence of tobacco use in Uganda with almost 1 in every 10 people in Uganda using tobacco products daily**.** Being older, male, having no formal education, residing in the east, north and western regions and having low BMI were significantly associated with daily tobacco use. This information provides a useful benchmark to the National Tobacco Control Program for the designing of public health interventions for the control and prevention of tobacco use in Uganda.

## Abbreviations

BMI, body mass index; GATS, Global Adult Tobacco Survey; NCD, Non Communicable Diseases; STEPS, stepwise approach to surveillance; UBOS Uganda Bureau of Statistics; UDHS, Uganda Demographic and Health Survey; WHO, World Health Organization

## Additional file

Additional file 1: Tobacco survey data. (XLS 5053 kb)

## References

[1] Riala K, Alaräisänen A, Taanila A, Hakko H, Timonen M, Räsänen P (2007). Regular daily smoking among 14-year-old adolescents increases the subsequent risk for suicide: the Northern Finland 1966 Birth Cohort Study. J Clin Psychiatry.

[2] Bhat VM, Cole JW, Sorkin JD, Wozniak MA, Malarcher AM, Giles WH (2008). Dose-response relationship between cigarette smoking and risk of ischemic stroke in young women. Stroke.

[3] Craig WY, Palomaki GE, Haddow JE (1989). Cigarette smoking and serum lipid and lipoprotein concentrations: an analysis of published data. BMJ.

[4] Castellsague X, Munoz N, Destefani E, Victora CG, Castelletto R, Rolon PA, Quintana MJ (1999). Independent and Joint effects of tobacco smoking and alcohol drinking on the risk of esophageal cancer in men and women. Int J Cancer.

[5] Alwan A. Global status report on noncommunicable diseases 2010: World Health Organization; 2011. Available from http://www.who.int/nmh/publications/ncd_report_full_en.pdf. [Accessed 18 Sep 2015].

[6] Ministry of Health, Uganda (2013). Tobacco related morbidity and mortality at Uganda Cancer Institute.

[7] World Health Organization. STEPwise approach to surveillance (STEPS) 2015. Available from: http://www.who.int/chp/steps/en/. [Accessed 22 Sep 2015].

[8] Uganda Bureau of Statistics (UBOS) and ICF International Inc (2012). Uganda Demographic Health Survey 2011. Kampala Uganda: UBOS and Calverton.

[9] Uganda Bureau of Statistics. National Population and Housing Census 2014 Revised Edition. Kampala: 2014. Available from http://unstats.un.org/unsd/demographic/sources/census/wphc/Uganda/UGA-2014-11.pdf. [Accessed 12 Jan 2016].

[10] Uganda Bureau of Statistics and ICF International Inc (2007). Uganda Demographic and Health Survey, 2006: Uganda Bureau of Statistics and Calverton.

[11] Uganda Bureau of Statistics (UBOS) and ICF International Inc (2001). Uganda Demographic and Health Survey 2000-2001. Kampala Uganda: UBOS and Calverton.

[12] Ministry of Health Uganda, Uganda Bureau of Statitstics, World Health Organisation Regional office for Africa, CDC Foundation, Centers for Disease Control. Global Adult Tobacco Survey: Executive Summary 2013 Uganda 2014 [cited 5th August 2015]. Available from: http://global.tobaccofreekids.org/files/pdfs/en/GATS_uganda_summary_2013.pdf.

[13] Guwatudde D, Mutungi G, Wesonga R, Kajjura R, Kasule H, Muwonge J (2015). The Epidemiology of Hypertension in Uganda: Findings from the National Non-Communicable Diseases Risk Factor Survey. PLoS One.

[14] Guwatudde D, Kirunda BE, Wesonga R, Mutungi G, Kajjura R, Kasule H, et al. Physical Activity Levels Among Adults in Uganda: Findings from a Countrywide Cross-Sectional Survey. J Phys Act Health. 2016; doi:10.1123/jpah.2015-0631. 10.1123/jpah.2015-0631 27172614

[15] Whitworth J, International Society of hypertension Writing Group (2003). 2003 World health Organization (WhO)/International Society of hypertension (ISh) statement on management of hypertension. J Hypertens.

[16] Longo M. Democratic Republic of Congo (Ville de Kinshasa) STEPS Survey 2005 Factsheet. 2005. Available from http://www.who.int/chp/steps/2005_DRC_FactSheet_EN.pdf. [Accessed 4 Dec 2015].

[17] Tesfaye F. Ethiopia (Butajira) STEPS Survey 2003 Fact sheet. 2003. Available from http://www.who.int/chp/steps/2006_Ethiopia_FactSheet_EN.pdf. [Accessed 7 Sep 2015].

[18] Ministry of Health Zambia, World Health Organization Country Office. Prevalence rates of the common non communicable disease risk factors in Lusaka district, Zambia 2008. 2008. Available from http://www.who.int/chp/steps/2008_Zambia_FactSheet_EN.pdf?ua=1. [Accessed 7 Sep 2015].

[19] Mayige M. Tanzania STEPS Survey 2012 Fact Sheet. 2012. Available from http://www.who.int/chp/steps/UR_Tanzania_FactSheet_2012.pdf?ua=1. [Accessed 7 Sep 2015].

[20] Damasceno AA. Mozambique STEPS Survey 2005 Factsheet. 2005. Available from http://www.who.int/chp/steps/2005_Mozambique_FactSheet_EN.pdf?ua=1. [Accessed 4 Dec 2015].

[21] Jha P, Chaloupka FJ (2000). The economics of global tobacco control. BMJ.

[22] Mpabulungi L, Muula A (2006). Tobacco use among high school students in a remote district of Arua, Uganda. Rural Remote Health.

[23] Gupta PC, Ray CS (2003). Smokeless tobacco and health in India and South Asia. Respirology.

[24] Foulds J, Ramstrom L, Burke M, Fagerström K (2003). Effect of smokeless tobacco (snus) on smoking and public health in Sweden. Tob Control.

[25] Phillips CV, Wang C, Guenzel B (2005). You might as well smoke; the misleading and harmful public message about smokeless tobacco. BMC Public Health.

[26] Mahalik JR, Burns SM, Syzdek M (2007). Masculinity and perceived normative health behaviors as predictors of men’s health behaviors. Soc Sci Med.

[27] Grunberg NE, Winders SE, Wewers ME (1991). Gender differences in tobacco use. Health Psychol.

[28] Sorensen G, Gupta PC, Pednekar MS (2005). Social Disparities in Tobacco Use in Mumbai, India: the roles of occupation, education, and gender. Am J Pub Health.

[29] Okidi JA, McKay A. Poverty dynamics in Uganda: 1992 to 2000. Economic Policy Research Centre Makerere, Kampala and School of Economics University of Nottingham. 2003. Available at SSRN: http://ssrn.com/abstract=1754443. [Accessed 7 Dec 2015].

[30] Najjumba-Mulindwa I. Chronic poverty among the elderly in Uganda: perceptions, experiences and policy issues. Conference ‘Staying Poor: Chronic Poverty and Development Policy’. Oxford, UK: University of Manchester; 2003.

[31] Davey Smith G, Hart C, Hole D, MacKinnon P, Gillis C, Watt G (1998). Education and occupational social class: which is the more important indicator of mortality risk?. J Epidemiol Community Health.

[32] Stead M, MacAskill S, MacKintosh A-M, Reece J, Eadie D (2001). “It’s as if you’re locked in”: qualitative explanations for area effects on smoking in disadvantaged communities. Health Place.

[33] Yach D, Bettcher D (2000). Globalisation of tobacco industry influence and new global responses. Tob Control.

[34] Albanes D, Jones DY, Micozzi MS, Mattson ME (1987). Associations between smoking and body weight in the US population: analysis of NHANES II. Am J Pub Health.

[35] Shimokata H, Muller DC, Andres R (1989). Studies in the distribution of body fat: III. Effects of cigarette smoking. JAMA.

[36] Potter BK, Pederson LL, Chan SS, Aubut J-AL, Koval JJ (2004). Does a relationship exist between body weight, concerns about weight, and smoking among adolescents? An integration of the literature with an emphasis on gender. Nicotine Tob Res.

[37] Chiolero A, Faeh D, Paccaud F, Cornuz J (2008). Consequences of smoking for body weight, body fat distribution, and insulin resistance. Am J Clin Nutr.

[38] Preston A (1990). Cigarette smoking-nutritional implications. Prog Food Nutr Sci.

